# DNA-based identification uncovers the illegal trade of sea cucumbers from Brazil

**DOI:** 10.1038/s41598-025-31971-6

**Published:** 2026-06-15

**Authors:** Auany Camila Fantinelli, Natália dos Santos, Carlos Egberto Rodrigues Junior, Daniel Eduardo Visciano de Carvalho, Rogério Caetano da Costa, Fabio Porto-Foresti, Ricardo Utsunomia

**Affiliations:** 1 Departamento de Ciências Biológicas, Faculdade de Ciências, Universidade Estadual Paulista (UNESP), Bauru, SP Brazil; 2 Instituto Brasileiro do Meio Ambiente e dos Recursos Naturais Renováveis (IBAMA), Brasília, DF Brazil; 3 Instituto Brasileiro do Meio Ambiente e dos Recursos Naturais Renováveis (IBAMA), São Paulo, SP Brazil

**Keywords:** DNA Barcoding, Forensic analysis, Illegal trade, Molecular taxonomy, Species identification, Ecology, Ecology, Environmental sciences

## Abstract

The illegal trade of sea cucumbers is widespread, driven by high international demand, particularly in Asia, where they are valued as culinary delicacies and for use in traditional medicine. Although domestic consumption in Brazil is limited, illegal harvesting for export is a growing concern, with unregulated fisheries posing a threat to local populations. In 2023, the Brazilian Institute of Environment and Renewable Natural Resources (IBAMA) seized dried sea cucumber specimens at Guarulhos International Airport. Using DNA barcoding with the Cytochrome C Oxidase I (COI) gene, we identified 18 specimens as *Holothuria grisea* and 22 as *Isostichopus badionotus*. Although both species are currently listed as “Least Concern” by the IUCN globally, the unregulated nature of this trade raises concerns about potential overexploitation, especially given the ecosystems they inhabit are increasingly vulnerable to habitat degradation and unsustainable practices. Additionally, the absence of *H. grisea* sequences in public genetic databases required us to collect fresh specimens to complete the analysis, underscoring the need for expanded molecular repositories. Open access to molecular repositories is a cornerstone of modern scientific progress, serving as a critical infrastructure for collaborative research. By providing unrestricted availability of standardized molecular data, these repositories not only prevent redundant investigations and optimize the use of research resources but also reinforce the robustness and reproducibility of scientific findings through independent validation. Furthermore, this democratization of knowledge levels the scientific playing field, enabling institutions of varying sizes and from diverse geographical locations to contribute equitably to the global research endeavor. Consequently, open access to these databases maximizes the return on public investment in science and significantly accelerates the pace of discovery in critical fields such as drug development and materials science. Our findings highlight the effectiveness of molecular tools in identifying illegally traded species, even in degraded forms, and emphasize the importance of stricter monitoring to protect biodiversity.

**Supplementary Information:**

The online version contains supplementary material available at https://doi.org/10.1038/s41598-025-31971-6.

## Introduction

Sea cucumbers, belonging to the phylum Echinodermata and the class Holothuroidea, are among the most significant marine resources, comprising approximately 1,250 species distributed globally^1^. In the Neotropical region, around 80 species of sea cucumbers have been cataloged^2,3^, with approximately 40 species of Holothuroidea recognized in Brazil^1^. Ecologically, these echinoderms play a crucial role as benthic organisms, exhibiting detritivorous feeding habits that contribute to the recycling and distribution of nutrients across marine sediments^4^. However, several species of sea cucumbers are currently at risk of extinction worldwide. This vulnerability is primarily driven by overfishing, degradation of marine habitats, and the impacts of climate change, all of which have significantly reduced their populations^3^.

Sea cucumbers are highly valued in Asia, where they are regarded as a culinary delicacy and an important component of traditional medicine, with a history spanning thousands of years^5–7^. The global demand for seafood and its derivatives continues to rise, positioning sea cucumbers as commercially valuable products with considerable economic value^8^. In some markets, they can fetch prices exceeding hundreds of dollars per kilogram, leading to the intensive harvesting of more than 82 species across nearly 70 countries^9^. However, these organisms are particularly vulnerable to overfishing due to their low mobility and the ease with which they can be collected, either manually during low tide or with trawling nets, making them easy targets for fishermen^10,11^.

In Brazil, the most encountered species of sea cucumbers are *Holothuria grisea* and *Isostichopus badionotus*, distributed along the northeastern and southern coasts of the country^12^. Although sea cucumber consumption is virtually non-existent in Brazil, illegal harvesting of these animals for export was already documented^13^. The dried and salted form of sea cucumber, known as *bêche-de-mer*, is a highly lucrative product in the seafood trade, often sold alongside shark fins^14,15^. Exporting dried specimens simplifies the process, as the reduced weight and volume make transportation more economical and efficient, allowing larger quantities to be shipped per load. Additionally, the dehydration process extends the shelf life of the product without refrigeration^14^. However, this also significantly alters the morphology of the sea cucumbers, making species identification and proper inspection procedures more difficult^3,14^.

The lack of regulation and formal declarations associated with this trade poses a serious threat to wild populations, potentially leading to a decline in population densities and even local extinctions^16^. For example, in China, the population of *Apostichopus japonicus*, a widely captured species, has significantly declined, prompting increased imports of sea cucumbers from other countries^13^. The depletion or extinction of sea cucumber populations could also result in the loss of undiscovered bioactive compounds with potential global health benefits^17^.

Accurate species identification is extremely important for several applications, especially for conservation and law enforcement. The illegal wildlife trade, a multibillion-dollar activity, is one of the main threats to marine biodiversity and involves several groups of marine organisms. Among them are sharks, heavily targeted for the fin trade, and sea cucumbers, which rank among the most exploited taxa^3,13,14^. DNA barcoding therefore proves to be an extremely useful technique, as the samples are rarely found in their natural state; they are often processed and dried as bêche-de-mer, making traditional morphological identification difficult and potentially leading to species mixture and mislabeling^11,16^.

DNA barcoding *sensu stricto* refers to the standardized 658-bp region of the mitochondrial cytochrome c oxidase I (COI) gene commonly used for animal identification, while in its broader sense the term may include any informative genetic marker used for taxonomic purposes. In this study, we rely on COI-based identification, a well-established and widely adopted method that follows the *sensu stricto* framework. By sequencing this fragment from an unknown sample and comparing it with a library of reference sequences, it becomes possible to obtain rapid and reliable species identifications^18,19^. This is particularly useful when working with taxonomically challenging groups or with processed products in which only small tissue pieces or degraded DNA remain^20,21^.

The accuracy of these identifications depends heavily on the quality of the reference databases. The Barcode of Life Data System (BOLD) was developed specifically for molecular taxonomy and provides curated barcode sequences accompanied by detailed metadata. GenBank, in contrast, is broader in scope and incorporates sequences from a wide range of studies, which means that metadata and taxonomic assignments can vary more in quality. Although different in purpose, both platforms are widely used and complement each other in DNA-based species identification^19^. An important feature of BOLD is its emphasis on linking sequences to voucher information, including specimen images and collection localities, which provides an additional level of confidence particularly relevant in legal or enforcement contexts^22,23^.

Because of these strengths, DNA barcoding has played a key role in revealing the true composition of the bêche-de-mer trade, frequently uncovering threatened species and cases of species substitution in marketed products^18,24,25^. In Brazil, the same approach has been essential for enforcement, bringing to light the sale of threatened elasmobranchs in situations where morphological identification of fillets was not possible^22,23^.

**Fig. 1:**
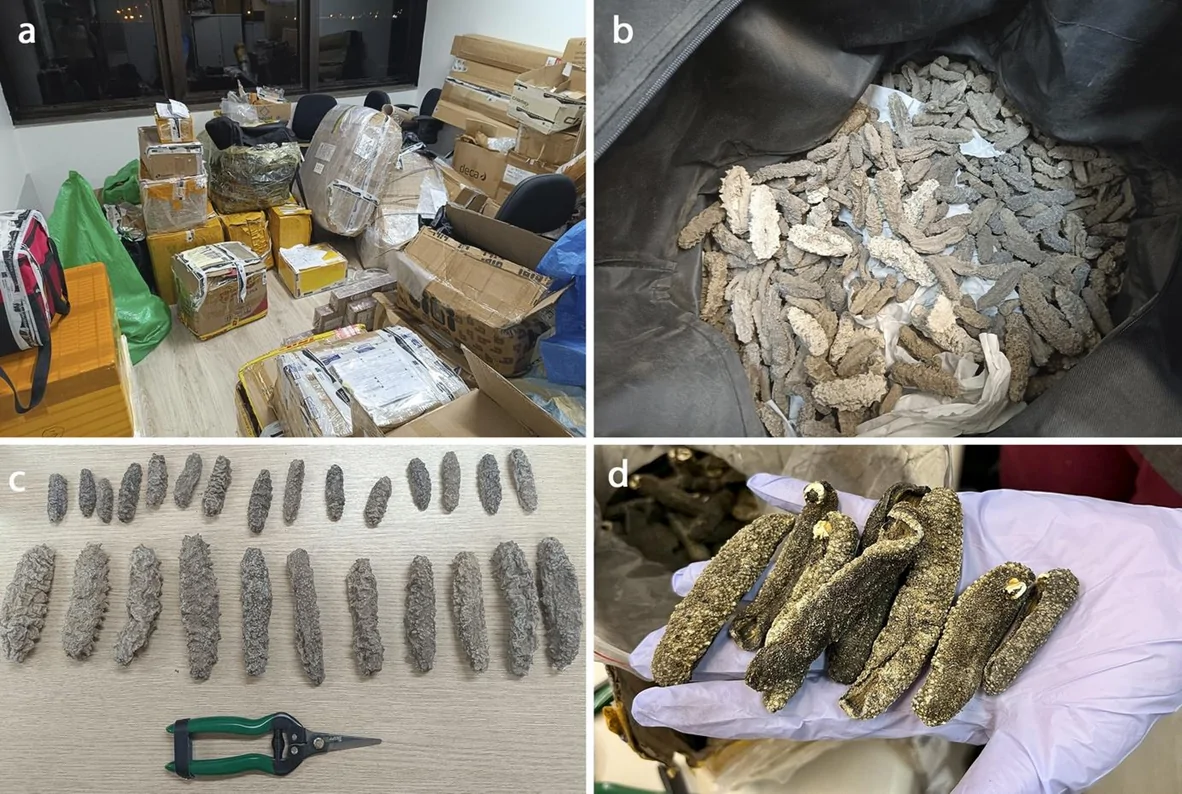
Seizure by IBAMA of boxes and suitcases, some containing sea cucumbers (a); a suitcase with sea cucumbers (b); different types of dried sea cucumbers (c, d).

In 2023, the Brazilian Institute of Environment and Renewable Natural Resources (IBAMA) seized sea cucumbers at Guarulhos International Airport (Guarulhos, São Paulo, Brazil). These specimens were found in both passenger luggage and packages sent from different cities in Southeastern Brazil (Fig. 1A). This study aimed to address key questions, including how many and which species of sea cucumbers are being illegally exported from Guarulhos International Airport (GRU)? Are these species native to the Brazilian coast? In addition, we assessed the conservation status of the identified species, as well as other holothurian species, based on the Brazil Red Book, the IUCN Red List, and CITES. In this context, molecular tools provide powerful means for accurately determining the taxonomic identity of these potentially exploited species.

To address these questions, we applied DNA barcoding on the seized specimens, targeting the mitochondrial Cytochrome C Oxidase I (COI) gene, which has been widely employed for species delimitation and identification, including sea cucumbers^18,20,26^. Furthermore, due to the lack of COI gene sequences for *H*. *grisea*, the predominant sea cucumber species along the Brazilian coast^27,28^, three live specimens were collected in Ubatuba (São Paulo, Brazil) and their COI sequences were characterized and submitted to the GenBank database (PQ468000-468002).

## Materials and methods

### Sample collection

Both dried and fresh samples were collected for this study. The dried samples, seized at GRU (Fig. 1b, c and d), were found inside boxes being exported from São Paulo to Italy (coded as PC229) and in the suitcases of two passengers attempting to illegally transport these animals (coded as PC206 and PC219). Each box and suitcase contained approximately 500 individual dried samples.

Two distinct morphotypes were recognized among the seized specimens based on external characteristics (Fig. 1c and d). To ensure both morphotypes were represented in the molecular analyses, a total of 40 sea cucumber specimens were randomly subsampled: 10 from PC206, 12 from PC219, and 18 from PC229. From these, 22 individuals of morphotype A and 18 individuals of morphotype B were selected for sequencing. Each individual was assigned a numeric code, and random selection was performed using a random number generator to prevent sampling bias.

In addition, three fresh samples of *Holothuria grisea* were collected from the rocky shores of Praia do Lamberto, in Ubatuba, São Paulo (23º30’00’’S 45º07’05’’W). Specimens were identified according to the taxonomic criteria established by Martins et al. (2012)^29^ to ensure accurate species determination. All samples were catalogued, photographed, and preserved in absolute ethanol before being deposited in the organism collection of the “Laboratório de Genômica e Conservação de Peixes” (LAGENPE), UNESP–Bauru/SP (Supplementary Table S1).

### DNA extraction and quantification

For DNA extraction, different techniques were tested to determine the most effective method for obtaining high-quality genomic DNA suitable for COI gene amplification. Comparisons were made between dry and rehydrated dry samples (which were soaked for three days in distilled water changed every 12 h) to assess the quality of the extraction.

**Fig. 2:**
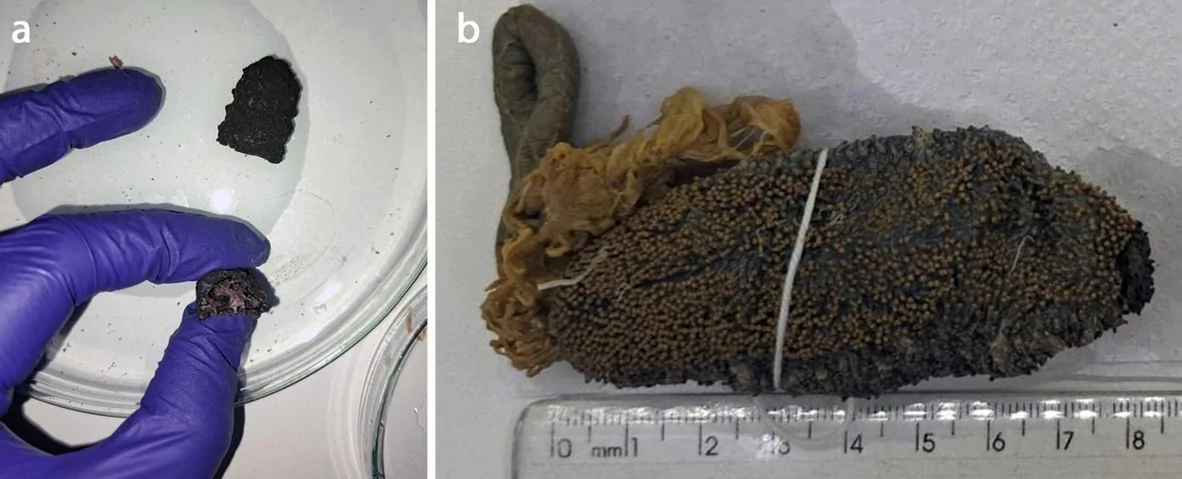
View of the part removed from the dried samples for DNA extraction (A) and fresh wild sample used for DNA extraction (B).

DNA extractions were carried out using the commercial “PureLink™ Genomic DNA Mini Kit” (ThermoFisher Scientific), following the manufacturer’s standard protocol. For the dried samples, tissue was obtained from the interior of the sea cucumber (Fig. 2a), while for the fresh samples, the animal’s intestine was used as the tissue source (Fig. 2b). DNA quantification was performed using the Qubit Fluorometer 2.0 (ThermoFisher Scientific), with the optimal DNA concentration for successful PCR reactions set between 1 and 2 ng/µl.

### Amplification and sequencing of COI gene

The primers used were those described by Ahmed et al.^30^, namely: COIPF 5’- CTAATGATAGGWGCCCCYGACATGGC − 3’ and COIPR 5’- CCTAGGTACCCRAAWGGYTCTTGCTT − 3’. These primers were aligned against COI sequences from eight different sea cucumber species (Supplementary Fig. 1), retrieved from the GenBank database, with the following accession numbers: KX384015, KX383993, JX683881, OP898058, FJ971395, OP898092, LC593268 and OP898065. The study by Ahmed et al. (2016) demonstrated the applicability of these primers across 18 Holothuroidea species, including several *Holothuria*, but none from *Isostichopus*. Considering that *H. grisea* and *I. badionotus* are the two most abundant species along the Brazilian coast^12^ and the most likely to occur in the seized material, we included both *Isostichopus* and *Holothuria* sequences in this in silico validation to confirm primer compatibility across the expected taxa.

The PCR was carried out with the following reaction components: 1x PCR buffer, 1,5 mM MgCl_2_, 200 µM of each dNTP, 0.1 µM of COIPF and COIPR primers, 0.5 U of Taq DNA polymerase (Invitrogen™ Platinum™) and 2 ng of template DNA. PCR reactions were performed in a ProFlex PCR System (Applied Biosystems) under the following cycling conditions: an initial denaturation at 94 °C for 4 min, followed by 30 cycles of 94 °C for 1 min; 58 °C for 1 min; 72 °C for 1 min, with a final extension at 72 °C for 10 min. PCR products were then analyzed by electrophoresis on a 2% agarose gel stained with GelRed Nucleic Acid Gel Stain (Biotium, Uniscience). Visualization was performed under ultraviolet light using a transilluminator, and images were captured with an OLYMPUS C 5060 camera.

Sequencing was performed using the BigDye Terminator v.3.1 Cycle Sequencing Ready Reaction kit (Applied Biosystems) with the same primers used in the PCR reactions. The sequencing was conducted on an ABI 3130-Genetic Analyzer (Applied Biosystems).

### Bioinformatic analysis

For each specimen, one forward and one reverse sequence were generated. The two strands were assembled and manually checked in Geneious Prime (Biomatters Ltd.)^31^ to obtain a consensus sequence. Base disagreements between forward and reverse reads were resolved by manually inspecting sequencing quality. Only high-quality consensus sequences were used in subsequent analyses, defined as those without ambiguous bases.

COI sequences of multiple species of sea cucumbers were obtained from the Barcode of Life Data System (BOLD) to ensure the inclusion of accurately curated and taxonomically validated data. Each consensus sequence generated in this study from seized specimens was queried against the BOLD public database using the standard search function. From the resulting matches, we retrieved the four Barcode Index Numbers (BINs) showing the highest similarity to each of our query sequences, thereby capturing the most relevant intraspecific and closely related interspecific variation. In addition, a COI sequence from *Enypniastes eximia* (order Elasipodida) was included as an outgroup.

The final dataset consisted of a combination of sequences from natural specimens, seized specimens, and sequences retrieved from GenBank and BOLD databases, resulting in a total of 493 COI sequences. These sequences were aligned using MUSCLE^32^. Reciprocal monophyly was used as the criterion for species identification, as performed in Alvarenga et al. (2021)^22^. Phylogenetic reconstruction was performed using maximum likelihood on the IQ-TREE webserver^33^. The nucleotide substitution model was selected using ModelFinder^34^ according to the Bayesian information criterion^35^, which identified TIM2e + + F + I + G4 as the best-fitting model. Branch support was calculated using 1,000 bootstrap replicates (UFBoot)^36^.

### Analysis of the conservation status of sea cucumbers

To better assess the conservation status and the level of exploitation faced by sea cucumber species, a manual search was conducted using three primary sources: the Brazil Red Book (https://www.gov.br/icmbio/pt-br/centrais-de-conteudo/publicacoes/publicacoes-diversas/livro_vermelho_2018_vol7.pdf), the International Union for Conservation of Nature Red List (IUCN Red List) at a global level (https://www.iucnredlist.org/search/stats), and the Convention on International Trade in Endangered Species of Wild Fauna and Flora (CITES) (https://www.gov.br/ibama/pt-br/assuntos/biodiversidade/cites-e-comercio-exterior/arquivos/legislacao/20240130_Anexo_18169051_Atualizacao_dos_anexos_Cites_portugues.pdf). These sources provide complementary perspectives on the vulnerability of sea cucumbers, encompassing both local and international conservation efforts.

## Results

### Sequencing and species identification

The results between the extractions from the dried and rehydrated individuals showed no significant differences between the two, so the dried samples were used for further analysis. PCR amplification and sequencing were successful for all 40 individuals, generating high-quality COI consensus sequences ranging from 290 to 480 bp in length (mean = 390.5 bp). Searches against the BOLD database returned matches corresponding to several holothurian species. For some specimens, the sequences showed the highest similarity with *H. edulis* (BIN: BOLD: AAC2033), *H. mammata* (BIN: BOLD: ABA2893), *H. atra* (BIN: BOLD: AAB2668), and *H. arguinensis* (BIN: BOLD: ABA2831). For other specimens, sequences matched *I. badionotus* (BIN: BOLD: AAW9189), *I. fuscus* (BIN: BOLD: ABA2119), *Isostichopus* sp. (BIN: BOLD: AAW9188) and *Stichopus hermanni* (BIN: BOLD: ACI2526). We retrieved sequences for all matched species, which were subsequently used for multiple sequence alignment, resulting in a final alignment of 899 characters, and then used in comparative analyses.

**Fig. 3:**
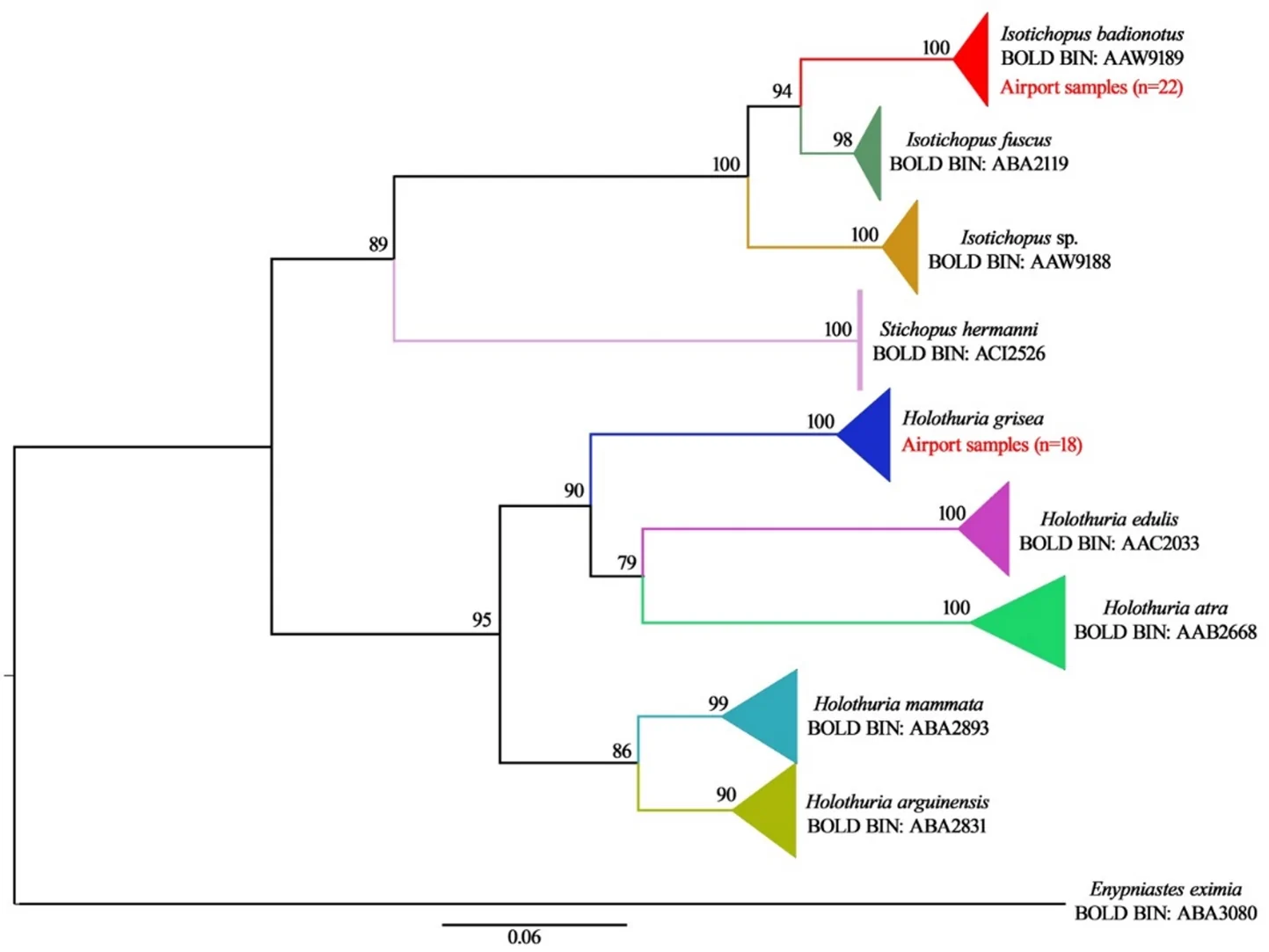
Phylogenetic species identification based on Maximum Likelihood analysis of the Cytochrome Oxidase Subunit 1 (COI) gene. Bootstrap support values from 1,000 replicates are shown. Branches are color-coded according to species names and their monophyletic status. Collapsed clades are labeled with the species name. Seized specimens are highlighted in red. The complete list of samples and the full phylogenetic tree are available in the supplementary Figure 2.

Phylogenetic reconstruction based on these sequences allowed the assignment of airport samples to two distinct Holothuroidea species (Fig. 3 and Supplementary Table S2): *Holothuria grisea* (*n* = 18) and *Isostichopus badionotus* (*n* = 22), two of the most abundant species along the Brazilian coast^12^. Phylogenetic analysis confirmed the species assignments, as our samples clustered with their respective species in well-supported monophyletic clades (bootstrap = 100). All *I. badionotus* samples formed a strongly supported monophyletic clade (branch support = 100; Fig. 3), and the *H. grisea* specimens clustered together similarly (branch support = 100), confirming the reliability of the taxonomic identifications.

### The relevance of DNA barcoding and genomic repositories for combating illegal trade and conservation status of sea cucumber species

To assess the conservation status of the species identified, we reviewed data from three primary sources: the Brazil Red Book, the IUCN Red List, and CITES. A detailed list of all identified species and their respective conservation statuses can be found in Supplementary Table S2. According to the Brazil Red Book, only one sea cucumber species, *Synaptula secreta*, is classified as Critically Endangered (CR). The IUCN Red List provides the conservation status of 371 sea cucumber species, with 244 classifieds as Data Deficient (DD) and 111 as Least Concern (LC). Nine species are considered Vulnerable (VU), including *Actinopyga echinites*, *A. mauritiana*, *A. miliaris*, *Apostichopus parvimensis*, *Bohadschia maculisparsa*, *Holothuria arenacava*, *H. fuscogilva*, *H. platei*, and *Stichopus herrmanni*. Additionally, seven species are listed as Endangered (EN): *Apostichopus japonicus*, *Holothuria lessoni*, *H. nobilis*, *H. scabra*, *H. whitmaei*, *Isostichopus fuscus*, and *Thelenota ananas*.

The CITES (Convention on International Trade in Endangered Species) database adopts a different classification system from the Brazil Red Book and IUCN. CITES organizes species into three appendices. Appendix I includes species at risk of extinction, where trade is allowed only under exceptional circumstances, generally for non-commercial purposes. Appendix II covers species not immediately threatened with extinction but whose trade requires regulation to prevent overexploitation. Appendix III lists species protected by specific countries, with international cooperation needed to control their trade. According to CITES, seven sea cucumber species are listed under these appendices. Six are included in Appendix II: *Holothuria fuscogilva*, *H. nobilis*, *H. whitmaei*, *Thelenota ananas*, *T. anax*, and *T. rubralineata*. One species, *Isostichopus fuscus*, is listed in Appendix I, restricting its trade to special conditions.

To bring this information together and connect it directly to enforcement, Table 1 summarizes the conservation status of key species and shows how DNA barcoding is specifically applied to monitor and protect them. For example, the table illustrates how DNA barcoding is crucial to uncovering the illegal domestic trade of *S. secreta* in Brazil and explains why it is a forensic priority for *I. fuscus*, a species prohibited from international trade. It also demonstrates the value of genetic identification for data-deficient species, such as *T. rubralineata*, where collecting reliable data on their occurrence is crucial to understanding how trade affects them.

**Table 1:** Conservation status and relevance for monitoring of sea cucumber species.

Species	Status (Brazil Red Book)	Status (IUCN Red List)	CITES appendix	Application of DNA barcoding for enforcement
*Synaptula secreta*	CR (Critically endangered)	Not Assessed	Not listed	Essential tool for detecting domestic illegal trade in Brazil, where the species is protected by national law
*Actinopyga echinites*	Not assessed	VU (Vulnerable) − 2010	Not listed	Identification of vulnerable species in international trade, aiding early warnings of overexploitation
*Actinopyga mauritiana*	Not assessed	VU (Vulnerable) − 2010	Not listed	Identification of vulnerable species in international trade, aiding early warnings of overexploitation
*Actinopyga miliaris*	Not assessed	VU (Vulnerable) − 2010	Not listed	Identification of vulnerable species in international trade, aiding early warnings of overexploitation
*Apostichopus parvimensis*	Not assessed	VU (Vulnerable) − 2010	Not listed	Identification of vulnerable species in international trade, aiding early warnings of overexploitation
*Bohadschia maculisparsa*	Not assessed	VU (Vulnerable) − 2010	Not listed	Identification of vulnerable species in international trade, aiding early warnings of overexploitation
*Holothuria arenacava*	Not assessed	VU (Vulnerable) − 2010	Not listed	Identification of vulnerable species in international trade, aiding early warnings of overexploitation
*Holothuria fuscogilva*	Not assessed	VU (Vulnerable) − 2010	II	Priority target for enforcement. International trade is regulated; DNA barcoding is required to verify the legality and origin of shipments
*Holothuria platei*	Not assessed	VU (Vulnerable) − 2010	Not listed	Identification of vulnerable species in international trade, aiding early warnings of overexploitation
*Stichopus herrmanni*	Not assessed	VU (Vulnerable) − 2010	Not listed	Identification of vulnerable species in international trade, aiding early warnings of overexploitation
*Apostichopus japonicus*	Not assessed	EN (Endangered) − 2010	Not listed	Identification of a globally threatened species, crucial for detecting and combating illegal markets
*Holothuria lessoni*	Not assessed	EN (Endangered) − 2010	Not listed	Identification of a globally threatened species, crucial for detecting and combating illegal markets
*Holothuria nobilis*	Not assessed	EN (Endangered) − 2010	II	High-risk target. High-value and endangered species. DNA barcoding is vital to authenticate the species in seized products and support legal actions against trafficking
*Holothuria scabra*	Not assessed	EN (Endangered) − 2010	Not listed	Identification of a globally threatened species, crucial for detecting and combating illegal markets
*Holothuria whitmaei*	Not assessed	EN (Endangered) − 2010	II	High-risk target. High-value and endangered species. DNA barcoding is vital to authenticate the species in seized products and support legal actions against trafficking
*Isostichopus fuscus*	Not assessed	EN (Endangered) − 2010	I	Maximum forensic priority. International commercial trade is prohibited. Its detection via DNA barcoding constitutes solid evidence of environmental crime
*Thelenota ananas*	Not assessed	EN (Endangered) − 2010	II	High-risk target. High-value and endangered species. DNA barcoding is vital to authenticate the species in seized products and support legal actions against trafficking
*Thelenota anax*	Not assessed	LC (Least Concern) − 2010	II	Demonstrates that CITES regulates species even without a high-risk assessment by IUCN. DNA barcoding verifies identity to ensure trade is legal and sustainable
*Thelenota rubralineata*	Not assessed	DD (Data Deficient) − 2010	II	For data-deficient species, correct identification via DNA barcoding is fundamental to generate real occurrence data and monitor the impact of trade

## Discussion

### DNA barcoding and the illegal and unregulated trade of sea cucumbers

The illegal trade of wildlife is a global issue that threatens biodiversity and can drive species to extinction, including those not yet described^18,19^. The multi-billion-dollar trade of exotic pets and food contributes to biodiversity loss, disease spread, and, in the case of pets, biological invasions^3,13,37^. This problem is exacerbated with food products, as many can be transported without special handling, complicating regulation^11,16^ and raising concerns about health and sanitation issues^20^. For example, the unregulated commercialization and illegal trade of elasmobranch products, like dried shark fins and rays, are well documented^21,23,38–40^. Similarly, other seafood products (including dried seahorses, shrimps, and sea cucumbers) are exploited without proper regulation, highlighting the urgent need for attention^24,41^.

In this study, despite the challenges typically associated with degraded or altered tissue samples, we successfully extracted DNA and PCR-amplified the COI gene from dried specimens illegally transported from Brazil, identifying them as *Holothuria grisea* and *Isostichopus badionotus*. This is particularly significant for species involved in the illegal wildlife trade, where processing often obscures key physical traits required for accurate identification^14^.

The commercialization of sea cucumbers has long been documented, and numerous COI gene sequences are available for different species in the NCBI database, which currently includes approximately 3,700 sequences representing more than 20 species, with *Holothuria atra*, *H. whitmaei*, and *Cucumaria frondosa* being the most abundant^19,25,42^. To the best of our knowledge, this is the first recorded instance of sea cucumber specimens being seized at an airport, raising concerns about this issue. These individuals were most likely captured in the wild, as there are no records of sea cucumber aquaculture in Brazil. The specimens were subsequently dried and prepared for illegal exportation. Although there are some initiatives to cultivate sea cucumbers in the state of Santa Catarina, these efforts remain incipient, despite the recognized potential for the development of this industry^25,43^.

Unregulated sea cucumber fisheries have already been reported in eastern Brazil, where dried specimens were found being transported to São Paulo and sold to specialized restaurants^16^. The present study further documents illegal shipping of *H. grisea* and *I. badionotus*, underscoring ongoing exploitation in the absence of effective regulatory measures. Our findings emphasize the need for continued monitoring to prevent overexploitation, particularly since both species, although abundant, are part of vulnerable ecosystems increasingly threatened by habitat degradation and unsustainable harvesting practices.

### Conservation status and gaps in knowledge

Our study highlights significant gaps in the conservation status of sea cucumber species. While the IUCN Red List identifies *H. grisea* as Least Concern (LC) and *I. badionotus* as having both local and national trade, these assessments are based on outdated data from 2010, with no information on recent population trends. This lack of current data complicates efforts to assess the true impact of the growing demand for these species. Additionally, discrepancies in the IUCN Red List, such as the misclassification of *I. badionotus* as confined to a small area in northeastern Brazil, further highlight the need for updated studies and accurate distribution maps.

The IUCN Red List indicates that 65% of the assessed Holothuroidea species are classified as Data Deficient (DD), reflecting insufficient knowledge about their populations and conservation needs. This knowledge gap hinders the development of effective conservation strategies and regulatory frameworks. The absence of COI gene data for *H. grisea* in public genetic databases also underscores the need for more comprehensive genetic studies, as we had to rely on fresh specimens to obtain reference sequences for this species.

The increasing global demand for sea cucumbers reflects patterns seen in the shark fin trade. Dried sea cucumbers and shark fins are widely traded in Asian markets and face growing conservation concerns^37,44^. As non-perishable products, such items are easily stored and transported, complicating regulation. Without proper monitoring, overexploitation of sea cucumbers could follow a trajectory similar to the sharp declines observed in shark populations due to the fin trade^37,45^.

The lack of consistent monitoring of sea cucumber populations and the outdated data in conservation databases further complicate efforts to control this trade. The IUCN Red List and CITES play crucial roles in regulating wildlife trade, but both require regular updates, including regional reassessments, to remain effective. In parallel, efforts should be made to expand databases such as NCBI and BOLD to include comprehensive genetic data for key species, thereby supporting more effective monitoring and management.

In Brazil, crimes against wildlife are classified as minor offenses under Brazilian legislation, with maximum penalties of up to two years, with or without fines. Specifically, environmental crime laws establish imprisonment from six months to one year and fines for those who illegally trade sea cucumbers, with aggravated penalties for offenses involving rare or protected species^46^. Moreover, Brazilian law allows for plea bargaining in such cases, enabling the extinction of penalties upon compliance with the conditions outlined in the agreement^47^.

## Conclusions

This study underscores the important role of molecular tools, such as DNA barcoding, in combating illegal and unregulated wildlife trade. By enabling the accurate identification of sea cucumbers–even in degraded forms like dried specimens–these tools help overcome the limitations of morphology-based identification. Our findings highlight the need for continuous monitoring and the expansion of genetic databases, such as NCBI and BOLD, to address existing gaps.

Ensuring the sustainable trade of sea cucumbers will depend on coordinated efforts between governments, conservation organizations, and local communities. Strengthening regulations, increasing public awareness, and encouraging sustainable harvesting practices are key to protecting these species and the ecosystems they support. Without collective action, sea cucumber populations could experience declines similar to those seen in sharks due to the fin trade, disrupting ecological balance. Future research must focus on closing gaps in our understanding of sea cucumber biology and trade patterns to develop more effective conservation strategies and promote the long-term sustainability of these vital marine resources.

## Supplementary Information

Below is the link to the electronic supplementary material.

Supplementary Material 1

Supplementary Material 2

## Data Availability

The datasets extracted and/or analyzed during the current study are available in the Genbank (NCBI) repository ( [https://www.ncbi.nlm.nih.gov/genbank/](https:/www.ncbi.nlm.nih.gov/genbank) ) and Barcode of Life Data System ( [https://boldsystems.org/](https:/boldsystems.org) ). Sequence data or seized that support the findings of this study were not deposited in GenBank or any other public repository, as the sequences are not linked to voucher specimens, as the samples were fully consumed during DNA extraction, leaving only photographic records as documentation. For this reason, we provide the FASTA file used in the analyses as supplementary material, including the sequences deposited in the databases (PQ468000–PQ468002), as well as individual images of each of the analyzed individuals.
