# Supplementary material for: DNA-based identification uncovers the illegal trade of sea cucumbers from Brazil
Source: Sci Rep. 2026 Jun 15;16:27221. doi: 10.1038/s41598-025-31971-6 (PMC13540865; doi:10.1038/s41598-025-31971-6)
Supplement: Supplementary file 1 [file 41598_2025_31971_MOESM1_ESM.pdf]

Supplementary Table 1: Registration numbers and photos of individuals

Registration Number - LAGENPE	Corresponding Number	Photo of the Individual
17327	PP1 - 3	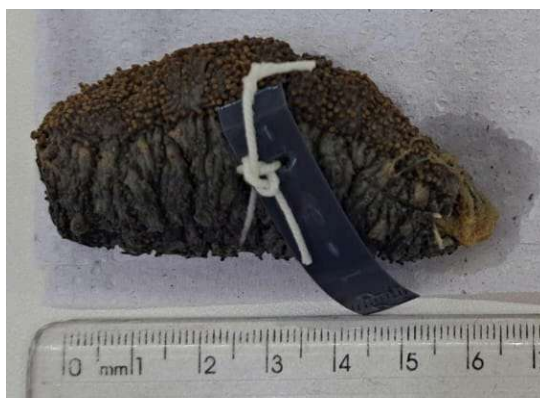
17329	PP2 - 1	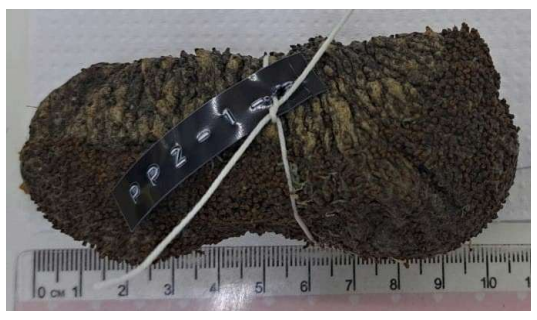
17337	PP4 - 2	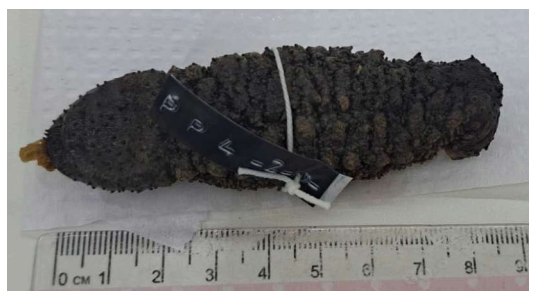
17340	PC 206 1	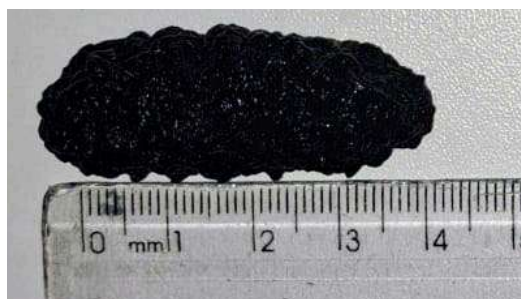
17342	PC 206 3	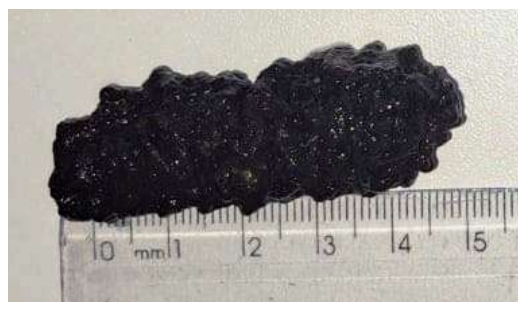
17343	PC 206 4	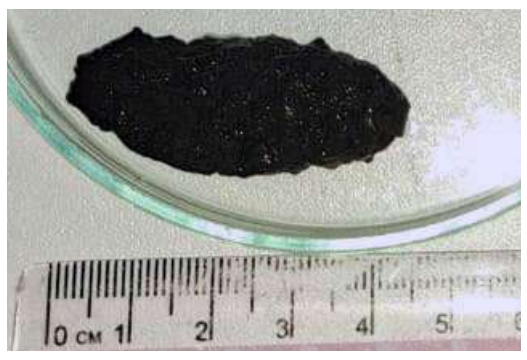
17345	PC 206 6	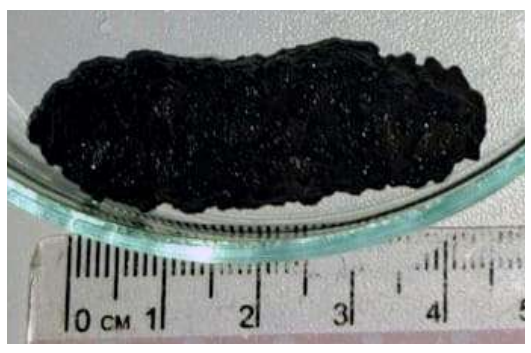
17346	PC 206 7	-

17347	PC 206 8	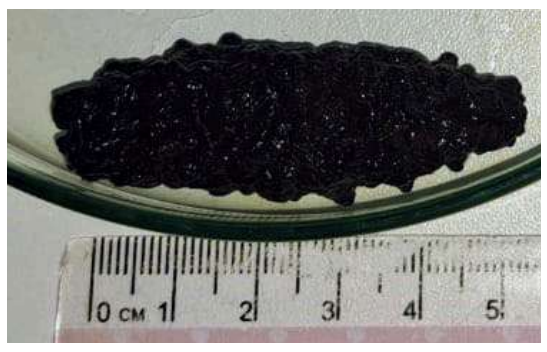
17349	PC 206 10	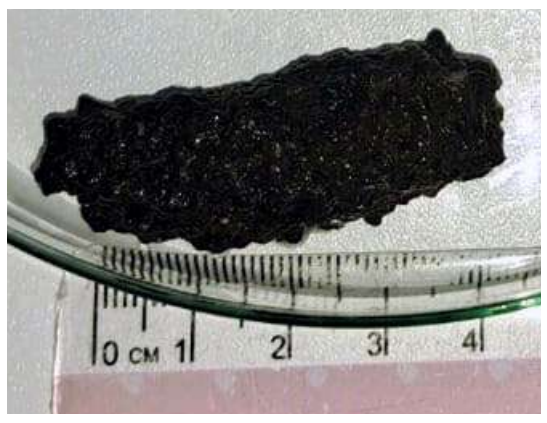
17350	PC 206 11	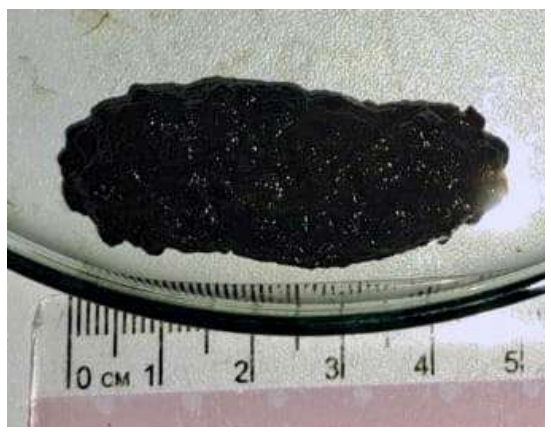
17351	PC 206 12	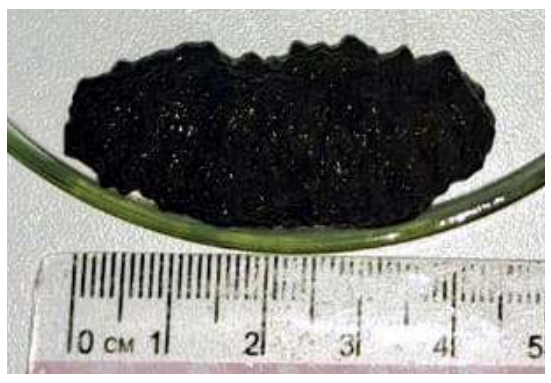
17394	PC 206 14	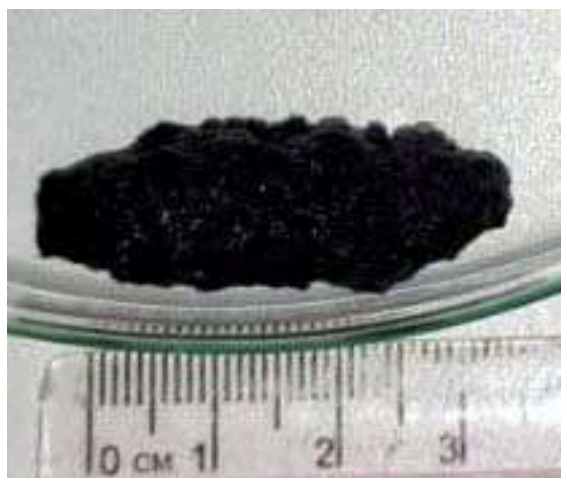
17395	PC 206 15	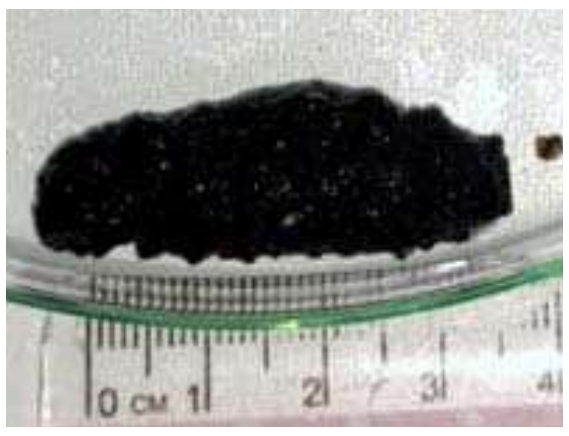
17356	PC 219 4	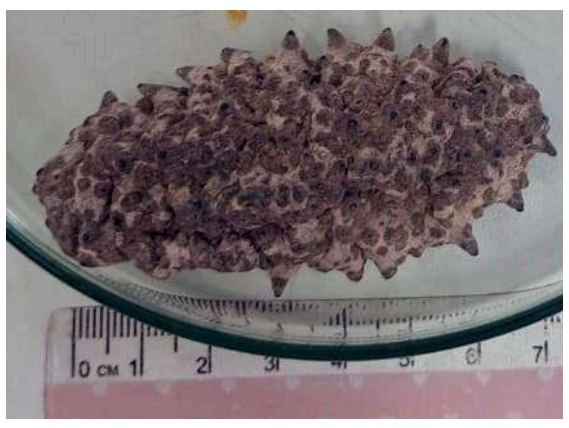

17357	PC 219 5	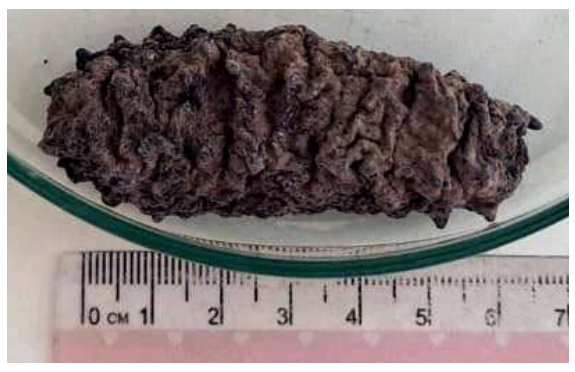
17358	PC 219 6	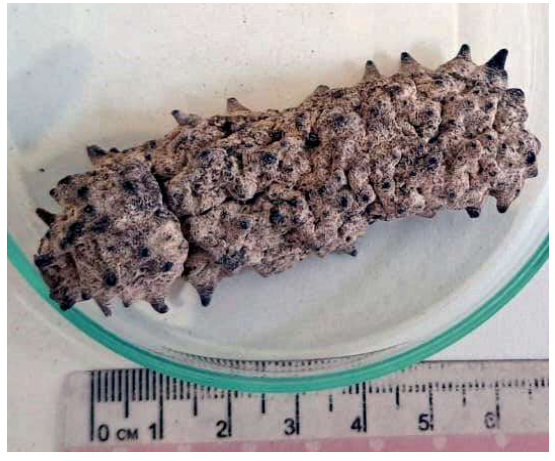
17361	PC 219 9	-
17363	PC 219 11	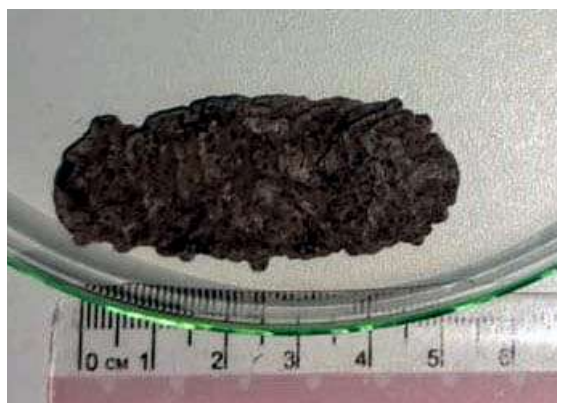
17364	PC 219 12	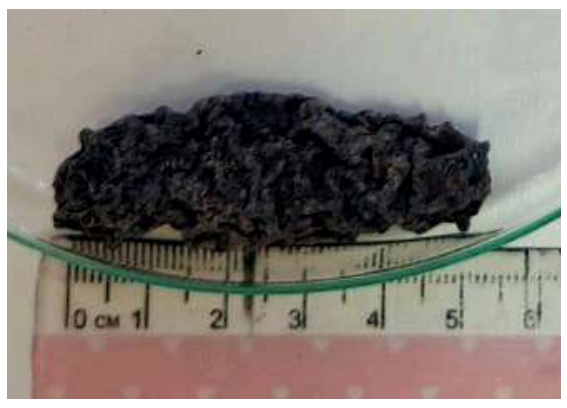
17365	PC 219 13	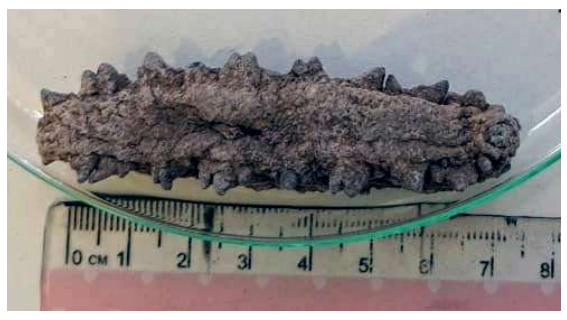
17366	PC 219 14	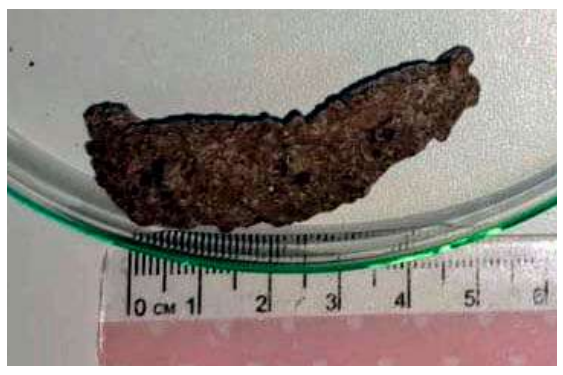
17367	PC 219 15	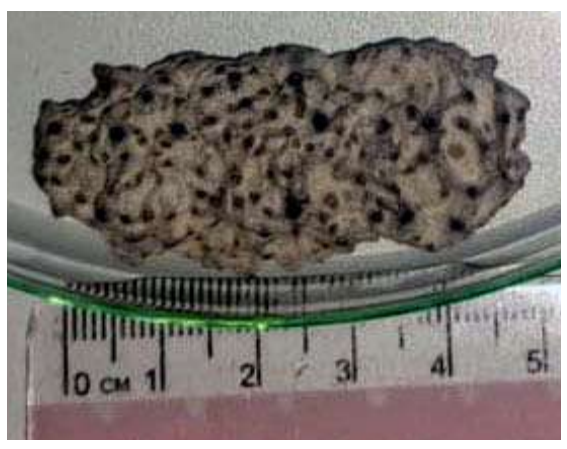

17368	PC 219 16	-
17369	PC 219 17	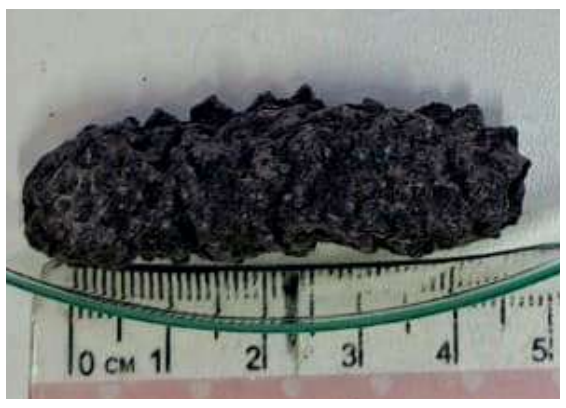
17372	PC 219 20	-
17374	PC 229 1	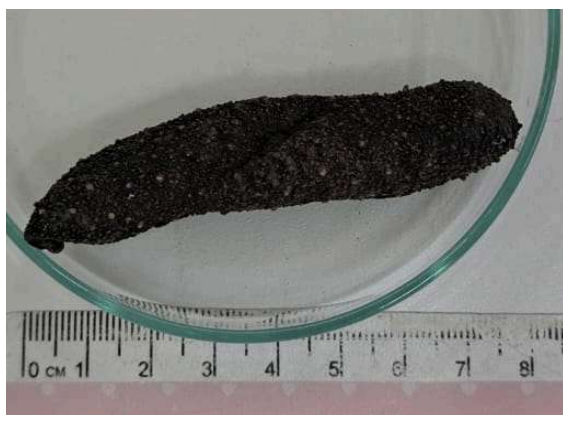
17375	PC 229 2	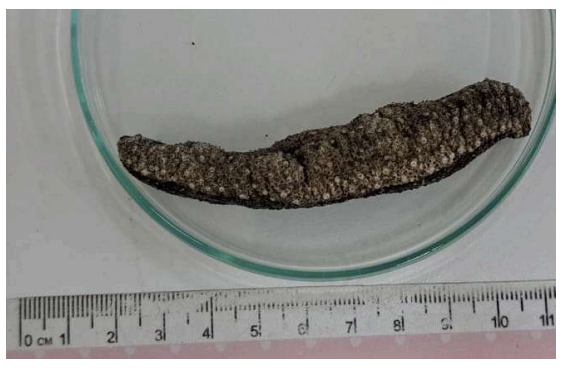
17376	PC 229 3	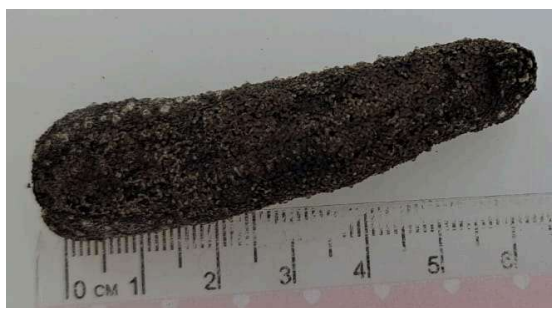
17377	PC 229 4	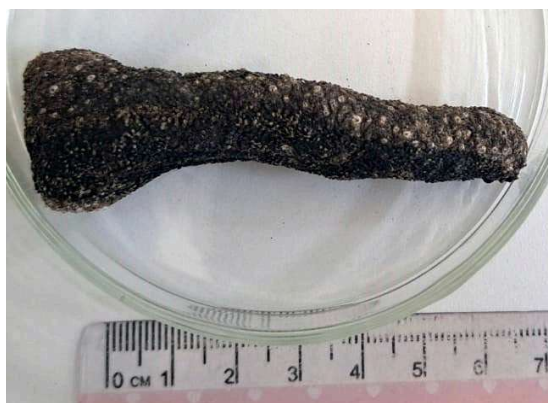
17378	PC 229 5	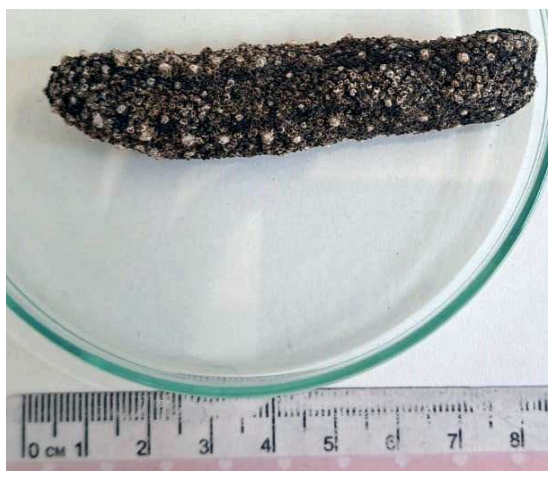

17379	PC 229 6	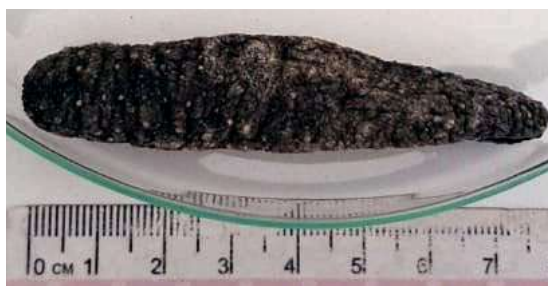
17382	PC 229 9	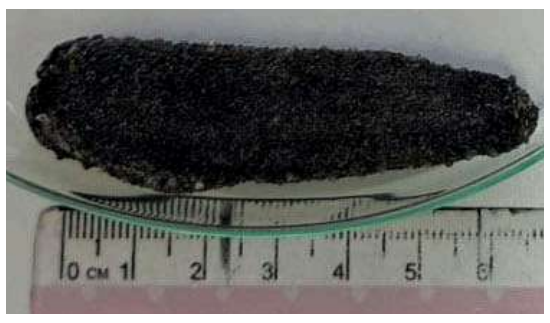
17383	PC 229 10	-
17384	PC 229 11	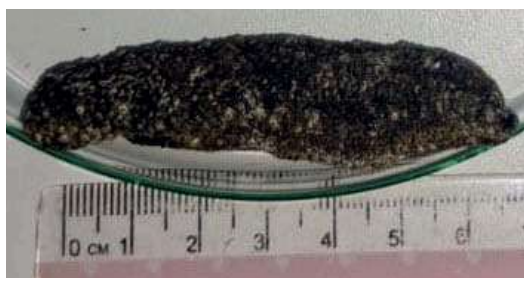
17386	PC 229 13	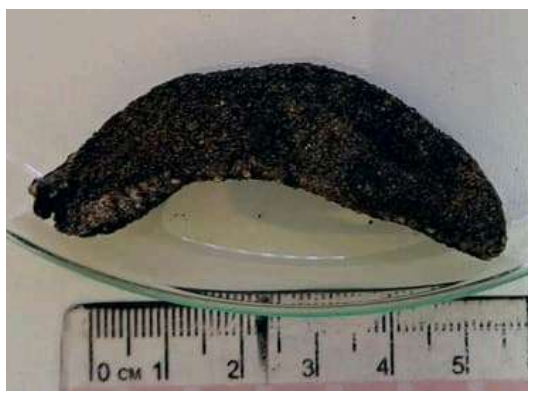
17387	PC 229 14	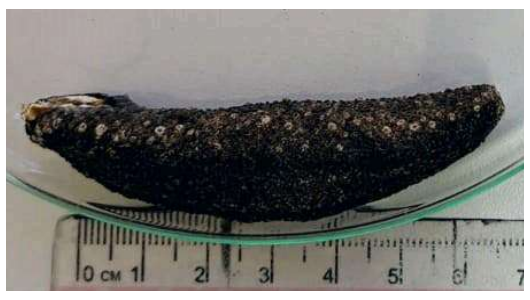
17388	PC 229 15	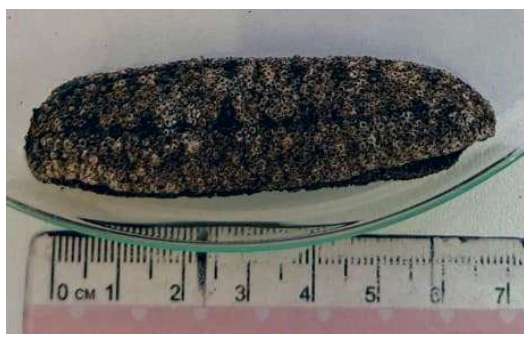
17389	PC 229 16	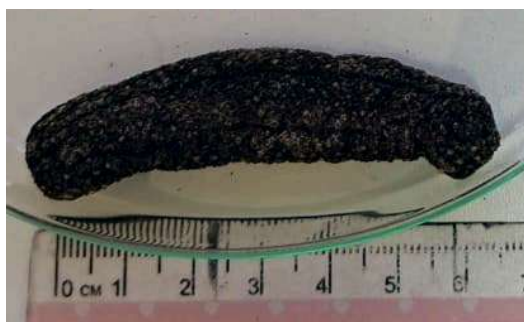
17390	PC 229 17	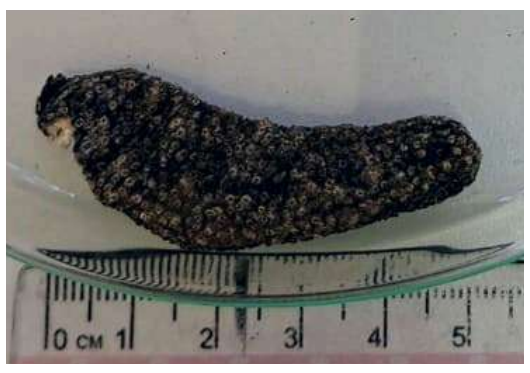

17391	PC 229 18	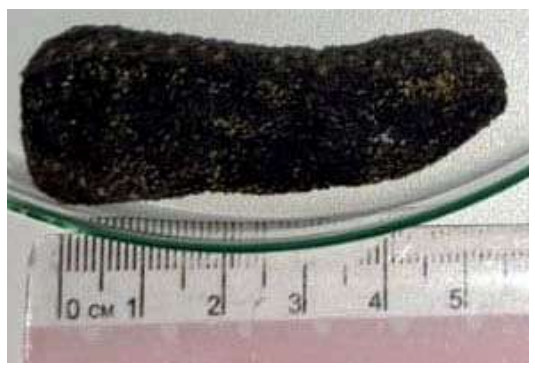
17392	PC 229 19	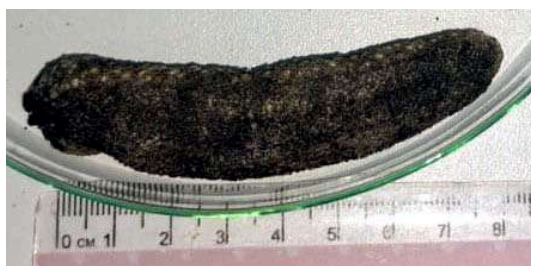
17393	PC 229 20	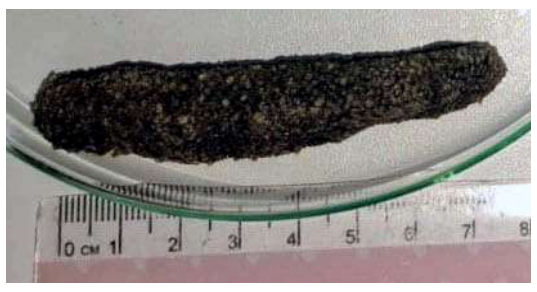

Most individuals had their morphology preserved in the form of photographs, however some specimens were not photographed. (-) individuals who do not have photographic records

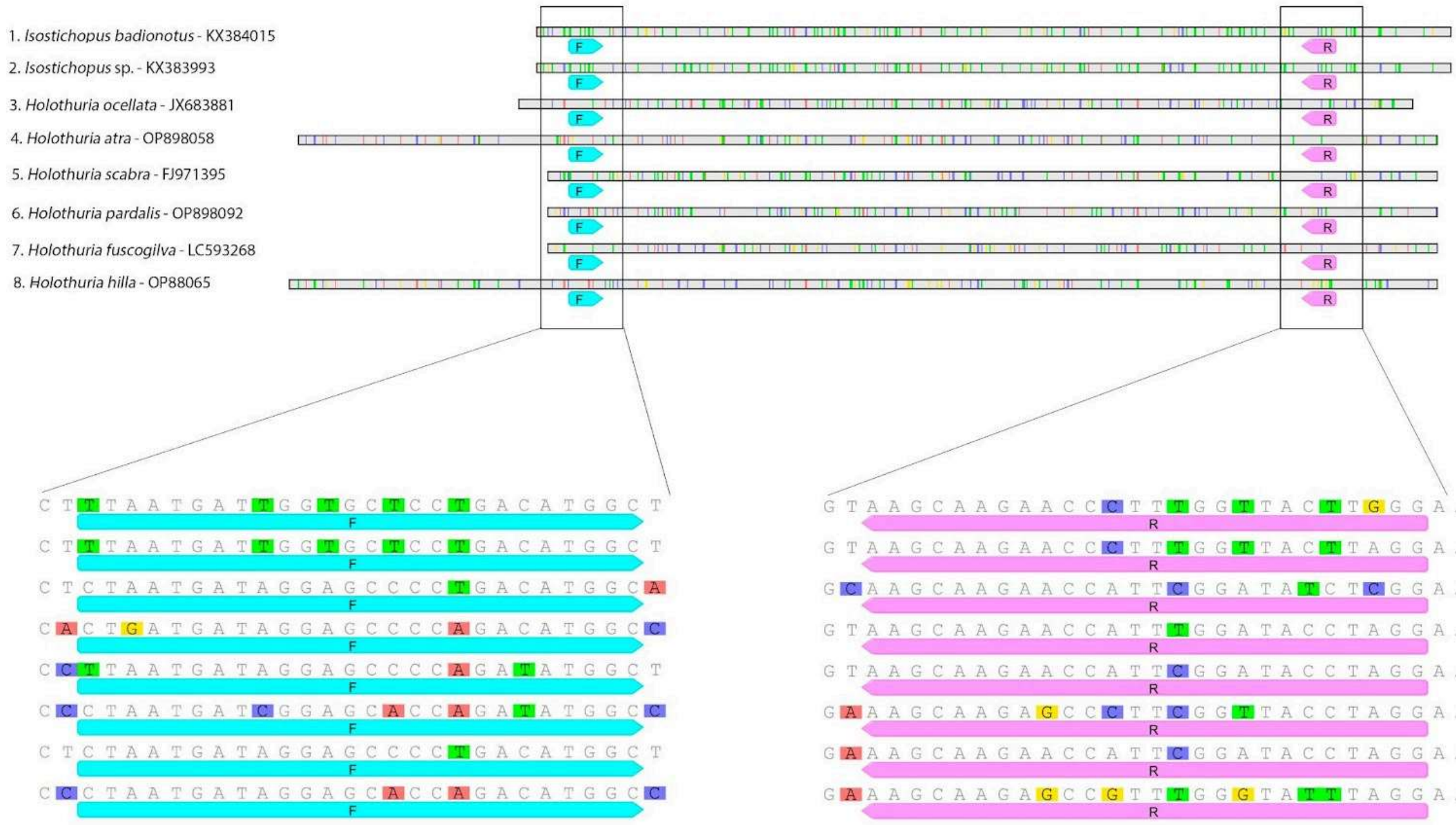

Supplementary Figure 1: Alignment of *Holothuria* and *Isostichopus* sequences with primers

Supplementary Table 2: Conservation Status Categories of Holothuroidea Species in Brazil Red Book, IUCN Red List and CITES (Convention on International Trade in Endangered Species)

Class	Order	Family	Scientific name	Conservation status		
				Brazil Red Book	IUCN Red List	CITES
Holothuroidea	Apodida	Synaptidae	*Synaptula secreta*	CR		
	Aspidochirotida	Holothuriidae	*Actinopyga agassizi*		LC	
			Actinopyga albonigra		DD	
			Actinopyga bacilla		DD	
			Actinopyga bannwarthi		DD	
			Actinopyga caerulea		DD	
			Actinopyga capillata		DD	
			Actinopyga crassa		LC	
			Actinopyga echinites		VU	
			Actinopyga flammea		LC	
			Actinopyga fusca		DD	
			Actinopyga lecanora		DD	

			Actinopyga mauritiana		VU	
			Actinopyga miliaris		VU	
			Actinopyga obesa		DD	
			Actinopyga palauensis		LC	
			Actinopyga serratidens		DD	
			Actinopyga spinea		LC	
			Actinopyga varians		DD	
			Argiodia maculata		DD	
			Bohadschia argus		LC	
			Bohadschia atra		DD	
			Bohadschia cousteaui		LC	
			Bohadschia koellikeri		DD	
			Bohadschia maculisparsa		VU	
			Bohadschia marmorata		DD	
			Bohadschia mitsioensis		DD	
			Bohadschia paradoxa		DD	

			Bohadschia similis		DD	
			Bohadschia steinitzi		DD	
			Bohadschia subrubra		DD	
			Bohadschia tenuissima		DD	
			Bohadschia vitiensis		DD	
			Holothuria aculeata		DD	
			Holothuria aemula		DD	
			Holothuria albifasciatus		DD	
			Holothuria albiventer		DD	
			Holothuria albofusca		DD	
			Holothuria altaturricula		DD	
			Holothuria annulifera		DD	
			Holothuria aphanes		DD	
			Holothuria arenacava		VU	
			Holothuria arenicola		DD	
			Holothuria		DD	

			arguinensis			
			Holothuria asperita		DD	
			Holothuria atra		LC	
			Holothuria austrinabassa		LC	
			Holothuria bacilla		DD	
			Holothuria brauni		DD	
			Holothuria cadelli		DD	
			Holothuria caparti		DD	
			Holothuria casoae		DD	
			Holothuria cavans		DD	
			Holothuria chilensis		DD	
			Holothuria cinerascens		LC	
			Holothuria coluber		LC	
			Holothuria conica		DD	
			Holothuria conusalba		DD	
			Holothuria coronopertusa		DD	
			Holothuria crosnieri		DD	
			Holothuria cubana		LC	

			Holothuria cumulus		DD	
			Holothuria dakarensis		LC	
			Holothuria dicorona		DD	
			Holothuria difficilis		LC	
			Holothuria discrepans		DD	
			Holothuria dofleinii		DD	
			Holothuria duoturricula		DD	
			Holothuria duoturriforma		DD	
			Holothuria dura		DD	
			Holothuria edulis		LC	
			Holothuria enalia		DD	
			Holothuria erinaceaus		LC	
			Holothuria excellens		DD	
			Holothuria exilis		DD	
			Holothuria flavocastanea		DD	
			Holothuria flavomaculata		LC	

			Holothuria floridana		LC	
			Holothuria foresti		DD	
			Holothuria forskali		LC	
			Holothuria fuligina		DD	
			Holothuria fuscocinerea		LC	
			Holothuria fuscogilva		VU	II
			Holothuria fuscopunctata		LC	
			Holothuria fuscorubra		DD	
			Holothuria glaberrima		LC	
			Holothuria glandifera		DD	
			Holothuria gracilis		DD	
			Holothuria granosa		DD	
			Holothuria grisea		LC	
			Holothuria hamata		DD	
			Holothuria hartmeyeri		DD	
			Holothuria hawaiiensis		LC	

			Holothuria helleri		LC	
			Holothuria hilla		LC	
			Holothuria horrida		DD	
			Holothuria huberti		DD	
			Holothuria imitans		LC	
			Holothuria immobilis		LC	
			Holothuria impatiens		DD	
			Holothuria inhabilis		LC	
			Holothuria inornata		DD	
			Holothuria insignis		LC	
			Holothuria isuga		LC	
			Holothuria integra		DD	
			Holothuria jousseaumei		DD	
			Holothuria kefersteinii		DD	
			Holothuria klunzingeri		DD	
			Holothuria kubaryi		DD	
			Holothuria languens		LC	
			Holothuria		LC	

			lentiginosa			
			Holothuria lessoni		EN	
			Holothuria leucospilota		LC	
			Holothuria lineata		DD	
			Holothuria longicosta		DD	
			Holothuria lubrica		LC	
			Holothuria macroperona		DD	
			Holothuria mactanensis		DD	
			Holothuria maculosa		DD	
			Holothuria mammata		LC	
			Holothuria mammosa		DD	
			Holothuria manningi		LC	
			Holothuria martensii		DD	
			Holothuria massaspicula		DD	
			Holothuria		LC	

			mexicana			
			Holothuria michaelseni		DD	
			Holothuria milloti		DD	
			Holothuria minax		DD	
			Holothuria moebii		LC	
			Holothuria modesta		DD	
			Holothuria multipilula		DD	
			Holothuria nigralutea		DD	
			Holothuria nobilis		EN	II
			Holothuria notabilis		DD	
			Holothuria occidentalis		LC	
			Holothuria olivacea		DD	
			Holothuria papillata		DD	
			Holothuria papillifera		DD	
			Holothuria paraprinceps		LC	
			Holothuria pardalis		LC	
			Holothuria parva		DD	

			Holothuria parvula		LC	
			Holothuria pervicax		LC	
			Holothuria platei		VU	
			Holothuria pluricuriosa		LC	
			Holothuria poli		LC	
			Holothuria portovallartensis		DD	
			Holothuria princeps		LC	
			Holothuria pseudofossor		LC	
			Holothuria pseudonotabilis		DD	
			Holothuria pulla		DD	
			Holothuria pyxis		DD	
			Holothuria pyxoides		DD	
			Holothuria rathbunii		LC	
			Holothuria remollescens		DD	
			Holothuria rigida		LC	
			Holothuria riojai		DD	
			Holothuria rowei		DD	
			Holothuria		DD	

			samoana			
			Holothuria sanctori		LC	
			Holothuria scabra		EN	
			Holothuria sinefibula		DD	
			Holothuria sinica		DD	
			Holothuria spinifera		DD	
			Holothuria squamifera		DD	
			Holothuria stellati		DD	
			Holothuria stocki		DD	
			Holothuria strigosa		DD	
			Holothuria submersa		DD	
			Holothuria sucosa		DD	
			Holothuria sulcata		DD	
			Holothuria surinamensis		LC	
			Holothuria suspecta		DD	
			Holothuria theeli		DD	
			Holothuria thomasi		LC	
			Holothuria tortonesei		DD	

			Holothuria tripilata		DD	
			Holothuria truncata		DD	
			Holothuria tuberculata		DD	
			Holothuria tubulosa		LC	
			Holothuria turriscelsa		LC	
			Holothuria turrisimperfecta		DD	
			Holothuria unica		DD	
			Holothuria vemae		DD	
			Holothuria verrucosa		LC	
			Holothuria vittalonga		DD	
			Holothuria whitmaei		EN	II
			Holothuria zacae		LC	
			Holothuria zihuatanensis		DD	
			Labidodemas americanum		LC	
			Labidodemas maccullochi		LC	
			Labidodemas		LC	

			pertinax			
			Labidodemas pseudosemperianum		DD	
			Labidodemas quadripartitum		DD	
			Labidodemas rugosum		LC	
			Labidodemas semperianum		LC	
			Labidodemas spineum		DD	
			Pearsonothuria graeffei		LC	
		Stichopodidae	*Apostichopus japonicus*		EN	
			Apostichopus johnsoni		DD	
			Apostichopus leukothele		LC	
			Apostichopus multidentis		DD	
			Astichopus multifidus		LC	
			Apostichopus nigripunctatus		DD	
			Apostichopus		DD	

			nipponensis			
			Apostichopus parvimensis		VU	
			Australostichopus mollis		LC	
			Eostichopus arnesoni		DD	
			Isostichopus badionotus		LC	
			Isostichopus fuscus		EN	III
			Isostichopus macroparentheses		DD	
			Neostichopus grammatus		DD	
			Neostichopus mollis		DD	
			Parastichopus nigripunctatus		DD	
			Parastichopus californicus		LC	
			Parastichopus regalis		LC	
			Parastichopus tremulus		DD	
			Stichopus anapinusus		DD	

			Stichopus chloronotus		LC	
			Stichopus ellipes		DD	
			Stichopus herrmanni		VU	
			Stichopus horrens		DD	
			Stichopus ludwigi		DD	
			Stichopus mollis		LC	
			Stichopus monotuberculatus		DD	
			Stichopus naso		LC	
			Stichopus ocellatus		DD	
			Stichopus paradoxus		DD	
			Stichopus pseudohorrens		DD	
			Stichopus noctivagus		LC	
			Stichopus quadrifasciatus		DD	
			Stichopus rubermaculosus		DD	
			Stichopus vastus		LC	
			Thelenota ananas		EN	II

			Thelenota anax		DD	II
			Thelenota rubralineata		DD	II
		Synallactidae	*Allopatides corrugatus*		DD	
			Allopatides dendroeides		DD	
			Amphigymnas bahamensis		LC	
			Amphigymnas multipes		DD	
			Bathyplotes baculosus		DD	
			Bathyplotes bigelowi		DD	
			Bathyplotes bongraini		DD	
			Bathyplotes cinctus		DD	
			Bathyplotes crenulata		DD	
			Bathyplotes golden-hindi		LC	
			Bathyplotes gourdoni		DD	
			Bathyplotes imperfectus		DD	

			Bathyplotes moseleyi		DD	
			Bathyplotes natans		LC	
			Bathyplotes phlegmaticus		DD	
			Bathyplotes pourtalesii		LC	
			Bathyplotes punctatus		DD	
			Bathyplotes roseus		DD	
			Bathyplotes rubicundus		DD	
			Bathyplotes spuma		DD	
			Bathyplotes sulcatus		DD	
			Bathyplotes tizardi		DD	
			Benthothuria cristata		LC	
			Benthothuria distorta		DD	
			Benthothuria funebris		LC	
			Benthothuria fusiformis		DD	
			Capheira mollis		LC	
			Capheira sulcata		LC	

			Hansenothuria benti		LC	
			Mesothuria abberviata		DD	
			Mesothuria bifurcata		DD	
			Mesothuria candelabra		DD	
			Mesothuria carnosa		DD	
			Mesothuria cathedralis		DD	
			Mesothuria connectens		DD	
			Mesothuria crebrapedes		DD	
			Mesothuria edwardensis		DD	
			Mesothuria expectans		DD	
			Mesothuria gargantua		LC	
			Mesothuria holothurioides		DD	
			Mesothuria incerta		DD	
			Mesothuria intestinalis		LC	

			Mesothuria lactea		LC	
			Mesothuria magellani		LC	
			Mesothuria marginata		DD	
			Mesothuria maroccana		DD	
			Mesothuria megapoda		LC	
			Mesothuria milleri		DD	
			Mesothuria multipes		LC	
			Mesothuria multipora		DD	
			Mesothuria murrayi		LC	
			Mesothuria oktaknemoides		DD	
			Mesothuria oktaknemus		DD	
			Mesothuria regularia		DD	
			Mesothuria roulei		DD	
			Mesothuria rugosa		LC	
			Mesothuria squamosa		DD	
			Mesothuria sufflava		DD	

			Mesothuria thomsoni		DD	
			Mesothuria triradiata		DD	
			Mesothuria verrilli		DD	
			Molpadiodemas atlanticus		DD	
			Molpadiodemas constrictus		LC	
			Molpadiodemas crinitus		DD	
			Molpadiodemas depressus		DD	
			Molpadiodemas epibiotus		LC	
			Molpadiodemas helios		DD	
			Molpadiodemas involutus		DD	
			Molpadiodemas morbillus		DD	
			Molpadiodemas neovillosus		DD	
			Molpadiodemas pediculus		DD	
			Molpadiodemas		LC	

			porphyrus			
			Molpadiodemas pustulosus		DD	
			Molpadiodemas translucens		DD	
			Molpadiodemas ustulatus		DD	
			Molpadiodemas villosus		LC	
			Molpadiodemas violaceus		LC	
			Paelopatides appendiculata		LC	
			Paelopatides atlantica		DD	
			Paelopatides confundens		DD	
			Paelopatides dissidens		DD	
			Paelopatides gelatinosa		DD	
			Paelopatides gigantea		DD	
			Paelopatides grisea		DD	
			Paelopatides illicitus		DD	

			Paelopatides insignis		DD	
			Paelopatides modestus		DD	
			Paelopatides mollis		DD	
			Paelopatides ovalis		DD	
			Paelopatides quadridens		DD	
			Paelopatides retifer		LC	
			Paelopatides solea		DD	
			Paelopatides suspecta		DD	
			Paelopatides mammillatus		LC	
			Paelopatides verrucosus		DD	
			Paroriza grevei		LC	
			Paroriza pallens		DD	
			Paroriza prohoi		DD	
			Paroriza verrucosa		DD	
			Perizona magna		DD	
			Pseudostichopus aemaulatus		DD	
			Pseudostichopus		DD	

			echinatus			
			Pseudostichopus elegans		LC	
			Pseudostichopus hyalegerus		LC	
			Pseudostichopus langeae		DD	
			Pseudostichopus mollis		DD	
			Pseudostichopus occulatatus		DD	
			Pseudostichopus papillatus		DD	
			Pseudostichopus peripatus		DD	
			Pseudostichopus profundi		DD	
			Pseudostichopus spiculiferus		DD	
			Pseudostichopus tuberosus		DD	
			Synallactes aenigma		LC	
			Synallactes alexandri		LC	
			Synallactes angustus		DD	

			Synallactes challengeri		LC	
			Synallactes chuni		DD	
			Synallactes crebrapapilla		DD	
			Synallactes crucifera		LC	
			Synallactes discoidalis		DD	
			Synallactes elongata		DD	
			Synallactes gilberti		LC	
			Synallactes horridus		LC	
			Synallactes laguardai		DD	
			Synallactes longipapillata		DD	
			Synallactes mollis		DD	
			Synallactes multivesiculatus		DD	
			Synallactes nozawai		LC	
			Synallactes pellucidus		DD	
			Synallactes rigidus		DD	
			Synallactes		DD	

			robertsoni			
			Synallactes sagamiensis		LC	
			Synallactes samyni		DD	
			Synallactes triplax		DD	
			Synallactes triradiata		LC	
			Synallactes viridilimus		DD	

Brazil Red List: CR - Critically Endangered/ IUCN Red List: DD - Data Deficient, EN - Endangered, LC- Least Concern, VU - Vulnerable/ CITES: II - Species that are not in danger of extinction, but may become so if exploitation incompatible with their survival is not avoided, III - Species protected by at least one contracting party, which has asked the others for their support in controlling international trade.
